# An integrated genetic and physical map of homoeologous chromosomes 12 and 26 in Upland cotton (*G. hirsutum *L.)

**DOI:** 10.1186/1471-2164-9-108

**Published:** 2008-02-28

**Authors:** Zhanyou Xu, Russell J Kohel, Guoli Song, Jaemin Cho, Jing Yu, Shuxun Yu, Jeffrey Tomkins, John Z Yu

**Affiliations:** 1 USDA-ARS, Southern Plains Agricultural Research Center, Crop Germplasm Research Unit, 2881 F&B Road, College Station, TX 77845, USA; 2 Chinese Academy of Agriculture Sciences, the Key Lab of Cotton Genetic Improvement of the Ministry of Agriculture, Cotton Research Institute, Anyang, Henan 455000, China; 3 Clemson University Genomics Institute, 51 New Cherry Road, Clemson, SC 29634, USA

## Abstract

**Background:**

Upland cotton (*G. hirsutum *L.) is the leading fiber crop worldwide. Genetic improvement of fiber quality and yield is facilitated by a variety of genomics tools. An integrated genetic and physical map is needed to better characterize quantitative trait loci and to allow for the positional cloning of valuable genes. However, developing integrated genomic tools for complex allotetraploid genomes, like that of cotton, is highly experimental. In this report, we describe an effective approach for developing an integrated physical framework that allows for the distinguishing between subgenomes in cotton.

**Results:**

A physical map has been developed with 220 and 115 BAC contigs for homeologous chromosomes 12 and 26, respectively, covering 73.49 Mb and 34.23 Mb in physical length. Approximately one half of the 220 contigs were anchored to the At subgenome only, while 48 of the 115 contigs were allocated to the Dt subgenome only. Between the two chromosomes, 67 contigs were shared with an estimated overall physical similarity between the two chromosomal homeologs at 40.0 %. A total of 401 fiber unigenes plus 214 non-fiber unigenes were located to chromosome 12 while 207 fiber unigenes plus 183 non-fiber unigenes were allocated to chromosome 26. Anchoring was done through an overgo hybridization approach and all anchored ESTs were functionally annotated via blast analysis.

**Conclusion:**

This integrated genomic map describes the first pair of homoeologous chromosomes of an allotetraploid genome in which BAC contigs were identified and partially separated through the use of chromosome-specific probes and locus-specific genetic markers. The approach used in this study should prove useful in the construction of genome-wide physical maps for polyploid plant genomes including Upland cotton. The identification of Gene-rich islands in the integrated map provides a platform for positional cloning of important genes and the targeted sequencing of specific genomic regions.

## Background

Cotton (*Gossypium *spp.) is the leading fiber crop worldwide and an important oil crop. Cotton is a diploidized allopolyploid species containing two subgenomes designated At and Dt. It is a model system to study polyploidization and post-polyploidization of plants. To develop tools essential for the genetic improvement of cotton and research in polyploid plant genetics, a number of genetic linkage maps have been developed [1-8]. As of this report, 6,921 specific loci including 440 quantitative trait loci (QTLs) [9], have been identified from 24 different genetic maps. Many traits of agronomic importance to cotton production have been mapped with these important genomic resources. In addition, a number of large-insert bacterial artificial chromosome (BAC) and plant transformation-competent binary large-insert plasmid clones (BIBAC) libraries have been constructed [10-13]. A large number of expressed sequence tags (ESTs), with a particular focus on fiber development, have been generated [14-16]. However, essential genomic tools are still in shortage, hindering further advances in such areas as DNA marker development for fine-scale mapping of genes and QTLs, genome-wide mapping of fiber ESTs, and large-scale genome sequencing.

Genome-wide integrated genetic and physical maps have provided powerful tools and infrastructure for advanced genomics research of human and other animal and plant model species. They are not only crucial for large-scale genome sequencing, but also provide powerful platforms required for many other aspects of genome research, including targeted marker development, efficient positional cloning, and high-throughput EST mapping [17]. Whole-genome physical maps have been constructed for *Arabidopsis thaliana *[18], rice [19], maize [20], and soybean [21]. However, no genome-wide physical map or chromosome contig map has been reported for any *Gossypium *species including Upland cotton (*G. hirsutum *L.). Genomics research of cotton has lagged behind that of other major crop plants such as maize, soybean, and wheat.

Upland cottons are thought to have formed about 1–2 million years ago by hybridization between an "A" genome *G. arboreum *or *G. herbaceum *and a "D" genome *G. raimondii *[22] or *G. gossypioides *[23]. The haploid genome size of Upland cotton has been estimated to be about 2,250 Mb [24]. Because genomes of the extant diploid species are only distantly related to those of cultivated tetraploid cottons, and Upland cottons account for more than 90% of world production, the International Cotton Genome Initiative (ICGI)[25] has proposed that the cotton research community develop a genome-wide physical map of Upland cotton (A_t _and D_t _subgenomes) that is based on the genetic standard 'TM-1' (inbred Upland germplasm line and one of the parents of the publically used mapping population TM-1 × 3–79) to facilitate integrated genomics research of cotton.

Allotetraploidy of Upland cotton presents a challenge in developing a robust integrated physical and genetic map and to specifically allocate contigs to their respective subgenomes. Chromosomes 12 and 26 have more genetic markers than the other pairs of chromosomes (Xu et al., unpublished) and were proved to be homoeologous chromosomes by genetic markers [5]. In this study, we test the feasibility of anchoring a wide diversity of existing genetic map data to a contig-based physical map and accurately assigning contigs to specific subgenomes and chromosomes. In doing so, all available genetically mapped cotton chromosome 12 and 26 markers associated with enough sequence to develop robust BAC library screening probes, were utilized, along with available BAC library resources. Having said this, we hypothesize that: 1) genetically mapped markers derived from ESTs and BAC-end sequences can be located in the cotton physical map by screening BAC libraries that have been fingerprinted and contiged, 2) that all other un-mapped EST data can also be anchored to the physical map, 3) that the information in the physical map can be markedly enhanced by annotating all anchored sequence, and 4) contigs can be accurately assigned to their subgenomes as well as to individual chromosomes.

## Results

### BAC library screening

A total of 287 and 207 DNA markers genetically mapped on chromosomes 12 and 26, respectively, were collected from 24 published genetic maps (Additional file 1). Of these, 166 and 128 markers were associated with enough known DNA sequence to be used for overgo primer design. After subjecting each sequence to overgo analysis, 162 (96.4%) and 120 (93.8%) overgo primers were designed for chromosomes 12 and 26, respectively. Of the overgo primers, 136 (83.9%) and 94 (78.3%) markers detected positive BAC clones. In total, 1,238 and 865 positive clones were selected from the three BAC libraries (Table 1) representing a 9.7X haploid coverage of the chromosomes. On average, there were 9.1 and 9.2 positive clones for each overgo primer associated with chromosomes 12 and 26, respectively, which is consistent with the 9.7X coverage estimate.

**Table 1:** Upland cotton BAC/BIBAC libraries used in the report.

Genotype	Mean insert size	No. of clones	Genome coverage	Vector type	Cloning site
TM-1	152 kb	76,800	5.2 ×	pECBAC1	*Hind*III
TM-1	130 kb	76,800	4.4 ×	pCLD04541	*Bam*HI
Maxxa	137 kb	2,603	0.15 ×	pCUGI-1	*Hind*III
Total	141 kb	156,203	9.7 ×		

In order to increase the genome coverage and cross-verify the contigs of the two chromosomes, all the positive BAC clones identified by non-repetitive markers were pooled for each chromosome and the pools were used as bulk probes to screen the three libraries again. There were 821 and 334 additional positive clones that resulted from the second round of selection for chromosomes 12 and 26, respectively. In total, there were 2,059 and 1,199 positive clones that were picked from the original BAC library plates for chromosomes 12 and 26, respectively. These clones were then re-arrayed into 35 96-well plates for fingerprinting.

### BAC fingerprinting and contig assembly

An initial total of 3,258 positive clones from the three BAC libraries were fingerprinted and the raw data was edited into FPC format via software "ABI-to-FPC" (written in C, unpublished). From the total number of clones, 241 clones (7.4%) were removed following fingerprint editing because they either failed in fingerprinting or had no inserts. In addition, 41 clones (1.3%) were ignored by the FPC [26] program during contig assembly because they contained five or fewer bands providing insufficient information to be included in the contig assembly. Thus, a final total of 2,976 clones were successfully fingerprinted and integrated into the FPC database. Between the chromosomes 12 and 26, 791 clones were shared between the two chromosomes (Additional file 2).

The FPC database of 2,976 BAC fingerprints was subjected to contig analysis using FPC software. The parameters employed in the contig assembly were: cutoff range 1e-35 to 1e-12 and a tolerance of 2. There were 220 and 115 BAC contigs and 5 and 7 singletons produced for chromosomes 12 and 26, respectively. The average number of DNA bands generated from each clone was 41 bands on a calculation using the whole FPC database. On average, each band counted for approximately 3359 bp, based on an overall average insert size for the three libraries of 141 kb (Table 1). There were 21,878 and 10,192 unique bands in the contigs for chromosomes 12 and 26, respectively. The sum total physical length of contigs was estimated to be 73.49 and 34.23 Mb for chromosomes 12 and 26, respectively.

### Genetic loci, contig number and genome characteristics

On chromosome 12, a total of 118 genetically mapped markers (28 SSR and 90 STS) were integrated into the physical map, which allowed for the anchoring of 220 contigs with an average of 1.9 contigs per marker (Additional file 2). Four of the 118 markers hybridized with single clones and the four clones could not be assembled into any contig at the low stringency of 1e-10. As a result, the four clones remained as four singletons and they were counted as four different loci on chromosome 12. In addition, 42 of the 118 markers hybridized with one contig, indicating a marker dense single region in the cotton genome, given the adequate level of genome coverage provided by the BAC clones, the data strongly indicated that this was a marker dense single-copy locus. On the other hand, 76 of the 118 markers hybridized with more than one contig, indicating multiple loci in the cotton genome. In summary, 46 (38.9%) of the 118 markers were single locus, and 76 (61.1%) remaining markers targeted multiple loci. Of the 220 contigs, 110 mapped only to chromosome 12 while another 110 also mapped to other regions of the genome.

On chromosome 26, sixty-five genetic markers (11 SSR and 54 STS) were anchored onto contigs or singletons, and 115 contigs were anchored by the 65 markers with an average about two contigs per marker. Eight of the 65 markers hybridized with single clones and could not be assembled into contigs or merged with other singletons at stringency 1e-10 (Additional file 3). As a result, the 8 clones remained as singletons and counted for 8 different loci on chromosome 26. In addition, 18 of the 65 markers hybridized with a single contig, indicating that these markers were also representative of a single marker-dense region in the cotton genome. Furthermore, 39 of the 65 markers hybridized with more than one contig, indicating that these markers had two or more loci in the Upland cotton genome. In summary, 26 (40%) of the 65 markers behaved as single copy, and 39 (60%) markers had multiple copies in the cotton genome. Of the 115 contigs, 48 were mapped specifically to chromosome 26, and 67 contigs also mapped to other regions of the genome.

Combining data on chromosomes 12 and 26, all the markers on the two chromosomes had an average of 1.8 contigs per marker. This result is consistent with the fact that Upland cotton has an allotetraploid genome.

### Homeology between chromosomes 12 and 26

Using marker-associated sequence comparisons via Blastn analysis, homeology between chromosome 12 and 26 was estimated to be 37.3% (Additional file 4). Based on physical mapping data, 67 contigs were shared between chromosomes 12 and 26 with the homeology estimated at about 40.0% between the two chromosomes in regard to extended genomic regions. Both analyses depict the allotetraploidy of the Upland cotton genome.

### Integrating cotton EST unigenes with the physical map

For chromosome 12, there were a total of 243 sequenced loci associated with 166 mapped markers and 77 BAC-end sequences. After the removal of redundant sequences, there were 224 unique sequences allocated to chromosome 12. At the time of this study, there were 24,137 fiber initiation unigenes, 20,169 elongation unigenes, 502 secondary cell wall deposition (SCWD) unigenes, and 19,160 non-fiber unigenes in the cotton community around the world. By use of sequence annotation via blastn with a matching criterion of at least 1e-30, there were 217 fiber initiation, 264 fiber elongation, 14 SCWD, and 214 non-fiber unigenes anchored to chromosome 12 (Additional file 5). Of the 224 mapped loci, 122 (54.5%) had an average of 4.03 unigenes per locus. Of those, 39 of the 122 loci contained only one unigene and the remaining 83 had more than one unigene. Because some ESTs were obtained at more than one plant growth stage, they were inadvertently counted more than once. A total of 401 fiber unigenes plus 91 non-fiber unigenes (492 EST unigenes) were anchored onto the integrated physical map of chromosome 12 after the removal of redundant sequences.

For chromosome 26, a similar strategy was used to map the cotton EST unigenes. There were a total of 141 sequenced loci allocated to chromosome 26. These included 127 genetically mapped sequences associated with EST derived markers and 14 BAC-end sequences. After removal of redundant sequence, there were 136 total annotated sequences assigned to chromosome 26. By use of blastn analysis with the same parameters for chromosome 12, there were 113 fiber initiation, 133 fiber elongation, 6 SCWD and 183 non-fiber unigenes that were integrated into the physical map (Additional file 6). In total, 207 fiber and 114 non-fiber unigenes (321 EST unigenes) along with 77 marker-based (EST and BAC-end) sequences were anchored to the physical map of chromosome 26 after removal of redundant sequence. Of the 77 sequence characterized loci, 27 had only one EST unigene while the remaining 50 had more than one EST unigene. On average, there were 4 EST unigenes allocated per locus.

In addition, a total of 16 and 13 function-verified ESTs were anchored by overgo hybridization into chromosomes 12 and 26, respectively.

**Figure 1:**
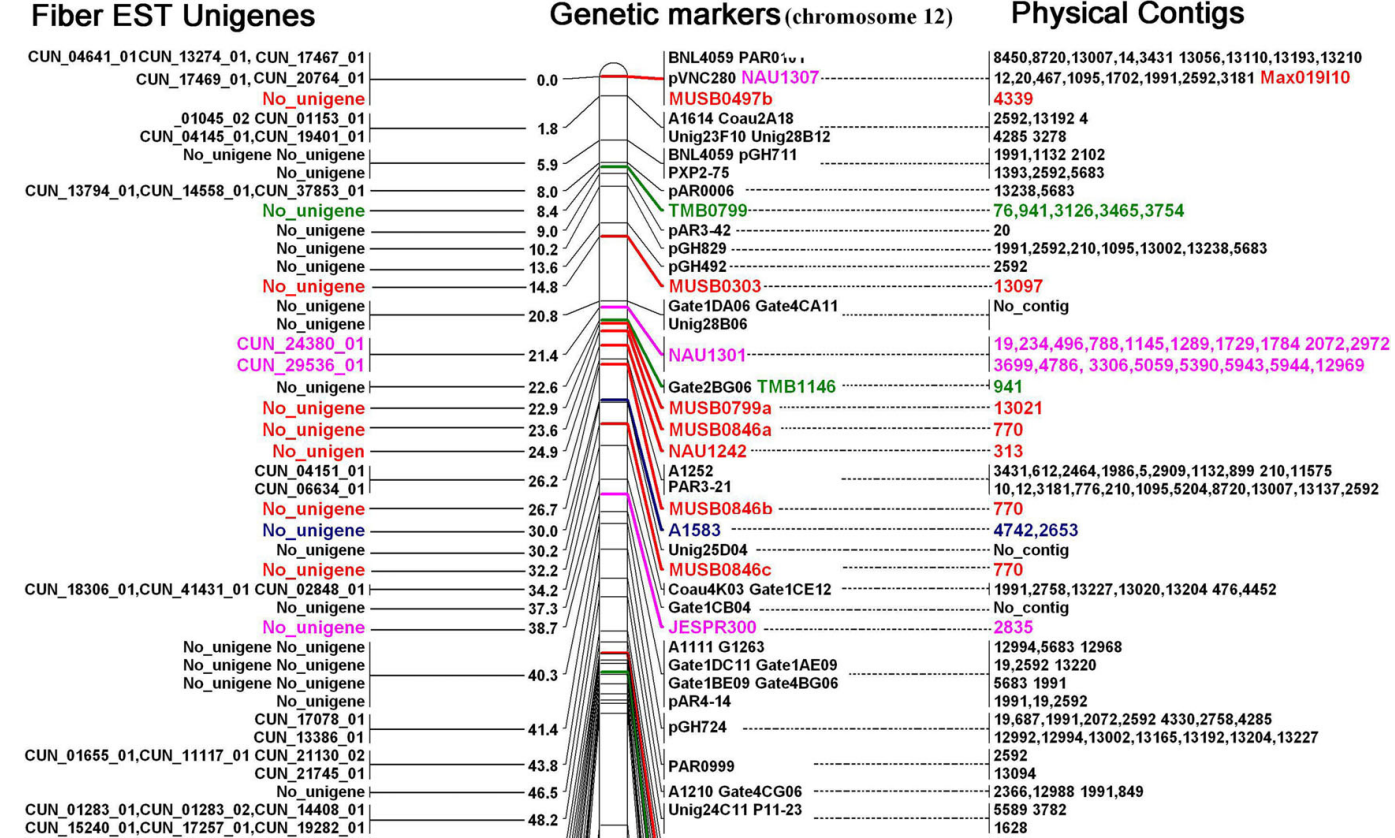
Integrated genetic, physical and transcript map of chromosome 12 (top part). Note: Three columns are displayed in the figure (left, middle and right). Left column shows the fiber EST unigenes anchored to the chromosome 12; Middle column shows the genetic map, and right column shows the contigs assembled from the positive clones to the genetic markers. The markers in black were used as backbone markers that were derived from an F_2 _mapping population (*G. hirsutum *race "palmeri" and *G. barbadense *acc. "K101); markers in red (MUSB) were from BAC-end sequence and genetic distance was from the RIL mapping population (*G. hirsutum *TM-1 × *G. barbadense *3–79); markers in green (TMB) were from BAC subcloned sequence and mapped by the TM-1 × 3–79 RIL population; the blue markers were from BC_1 _mapping population ('Guazuncho 2' × 'VH8-4602'). Markers in pink at the bottom of the figure were from BC_1 _mapping population (TM-1 × (TM-1 × Hai7124). CUN stand for Cotton Unigene Number that was used in the original paper [16]. This figure shows the upper part of the whole figure, for the full image please see additional file 7.

**Figure 2:**
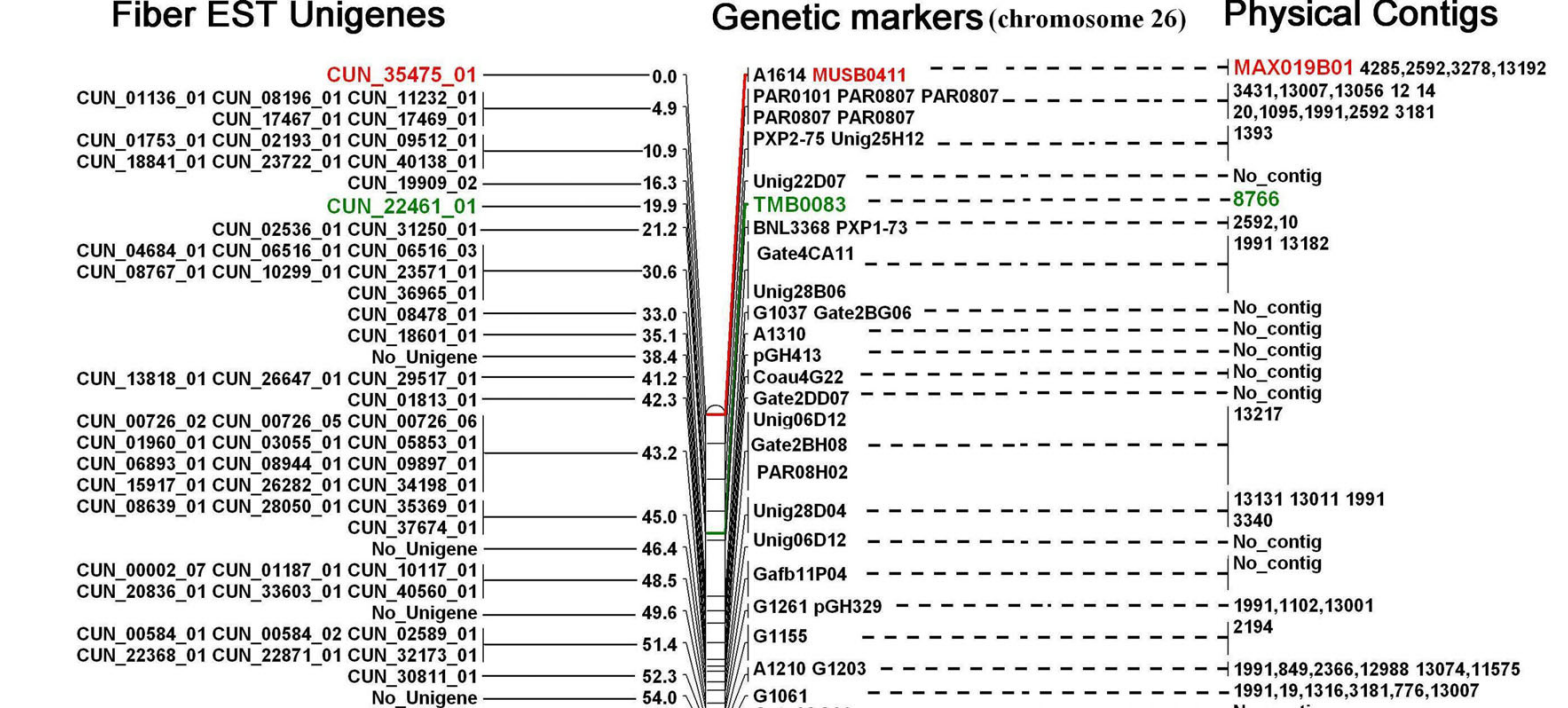
Integrated genetic, physical and transcript map of chromosome 26 (top part). All legends are same as described for Figure. 1. This figure shows the upper part of the whole figure, for the full image please see additional file 8.

The integrated maps of chromosomes 12 and 26 are shown in part (Figures 1 and 2) and in whole (Additional files 7 and 8). There are 492 and 321 EST unigenes exclusively located on chromosomes 12 and 26 respectively. However, eighty-five unigenes (20.9%) are also shared between these two chromosomes, indicating the presence of functional homeologs.

### Unigene distribution and gene-rich islands on chromosomes 12 and 26

EST unigenes were unevenly distributed on chromosomes 12 and 26. To analyze this statistically, we partitioned each linkage group into intervals of 10 cM in length. On the basis of the total number of EST unigenes per interval, the Poisson probability distribution function was used to identify bins that contained significant (p < 0.001) excesses or deficiencies of various classes of EST unigenes. As to the fiber gene distribution on chromosome 12, a total of ten intervals were identified, and 5 of them were far larger than the average number and 5 smaller than the average. By outlier analysis, 4 intervals (p < 0.001) were well outside the bulk of the data, representing distant gene-rich islands (Table 2). As to the non-fiber

**Table 2:** Distribution of fiber and non-fiber EST unigenes on chromosomes 12 and 26.

Genetic distance (cM)	Chromosome 12 No. fiber unigenes	No. non-fiber unigenes	Chromosome 26 No. fiber unigenes	No. non-fiber unigenes
0.0–9.9	19	13	6	8
10.0–19.9	10	7	7	1
20.0–29.9	17	9	4	1
30.0–39.9	12	4	10	6
40.0–49.9	42*	30	28*	13
50.0–59.9	50*	19	14	8
60.0–69.9	0	0	5	7
70.0–79.9	11	3	8	6
80.0–89.9	3	1	3	2
90.0–99.9	3	1	50*	25*
100.0–109.9	9	5	0	0
110.0–119.9	5	4	21	20*
120.0–129.9	6	2	7	3
130.0–139.9	32	14	12	5
140.0–149.9	49*	21	9	15
150.0–159.9	8	8	11	6
160.0–169.9	11	3	4	51**
170.0–179.9	7	7	0	0
180.0–189.9	62**	25	0	0
190.0–199.9	18	14	0	1
200.0–209.9	11	5	4	1
210.0–219.9	4	2	1	0
Others	12	17	3	4
Total	401	214	207	183
MOTV^a^	38.5	30.5	23.0	18.5
EOTV^b^	58.0	47.0	35.0	29.0

*: Numbers underlined and bold with one star are mild outliers; numbers underlined and bold with two stars are extreme outliers.

^a^: MOV stands for Mild Outlier Threshold Value; ^b^: EOTV stands for Extreme Outlier Threshold Value.

EST unigenes on chromosome 12, a total of 6 intervals had either more or less than the average number of unigenes by the Poisson probability distribution analysis (p < 0.001). However, none of them reached to an outlier. Therefore, there were no non-fiber gene-rich islands on chromosome 12. A similar analysis was applied to chromosome 26 where there were 7 fiber ESTs and 5 non-fiber EST intervals (p < 0.001) that had more or less than the average number of EST unigenes. There were two fiber gene-rich (p < 0.001) and three non-fiber gene-rich islands/outliers (p < 0.001) in chromosome 26 (Table 2). The total numbers of cotton unigenes anchored and gene-rich islands on the integrated map of chromosomes 12 and 26 were summarized in Table 3.

**Table 3:** Summary of the integrated genetic, physical and transcript map of chromosomes 12 and 26.

	Chromosome 12	Chromosome 26
Total markers	287	207
Sequenced markers	166	128
Anchored markers	118	65
Single-locus markers	46	26
Positive BAC clones	2,059	1,199
Shared clones	791	791
Assembled contigs	220	115
Contigs anchored on one chromosome	110	48
Contigs shared between 12 and 26	67	67
Physical length (Mb)	73.5	34.2
Anchored fiber EST unigenes	401	207
Total anchored EST unigenes	492	321
Gene-rich islands*	4	5

*Gene-rich islands were identified by the number of EST unigenes per 10 cM interval; distribution pattern was tested by Poisson probability (p < 0.001) and gene-rich islands were confirmed by outlier statistic standard analysis.

## Discussion

### Possibilities for a consensus map of the Upland cotton genome

Although several genetic maps have been constructed, most of them used different mapping populations with different population sizes. As a result, the genetic markers were often mapped at different genetic distances in different maps. This makes it difficult to study gene distribution, evolution, and map-based cloning between populations. This level of uncertainty also complicates the use of genetic markers to allocate contigs to chromosomes. In this report, contig 183 containing EST-derived SSR marker NAU1119 and contig 8766 with BAC-end derived SSR marker TMB0083, cannot be precisely merged into the saturated genetic map [5] because they were mapped with different populations. The genetic distance of a given marker derived from different mapping populations or from the same cross, but with different population sizes, is often significantly different. Even if population parameters are the same, differences in the number of genetic markers used, can cause variations in genetic distance. For example, marker NAU1119 was mapped on chromosome 26 but at different locations; 123.6 cM and 207.3 cM, in the same BC_1 _mapping population of TM-1 × (TM-1 × Hai7124) [8]. The optimistic point is that even though the genetic distances from different maps were different, the order of the markers in different maps showed an almost perfect colinearity [4]. Recently, more research groups are beginning to exploit a permanent mapping population based on the RIL mapping population of TM-1 × 3–79. However, many markers located on other maps have not yet been integrated into the TM-1 × 3–79 RIL. In order to use the previously mapped genetic markers to anchor contigs to chromosomes, a consensus genetic map based on the RIL population is needed. This could be achieved *in silico *by mapping a subset of common markers for each chromosome in different populations. Using this approach, it may be possible to obtain a consensus for marker order and recombination distances. On the other hand, integration of genetic and physical maps also helps align different markers from different linkage maps into a consensus genome map. A large contig having two or more markers in different maps could be used as evidence to align two or more linkage groups into one consensus map.

### Genetic distance versus physical distance in cotton

In our study, there was a lack of direct association between genetic distance and physical distance. A previous report in cotton showed that the overall average genetic distance between consecutive loci is 1.72 cM with a range of 1.44 cM (chromosome 8) to 2.23 cM (chromosome 2) [5]. Based on genetically mapped markers, an average interval of ~606 kb between two neighboring markers was expected using a genome size estimate of ~2700 Mb. If the genome size estimated to be ~2118 Mb [12], an interval of less than 475 kb between two markers would be expected. By our initial first glimpse into physical vs. genetic distance in cotton, one cM would account for an expected range of ~276 kb (genome size 2,118 Mb) to ~352 kb (genome size 2700 Mb). In this study, contig 274 contained two STS markers; Unig22H11 and Gafb28I12, with respective genetic position of 56.3 and 54.1 cM. The two markers are associated with fiber elongation ESTs and located in two overlapped BAC clones (CBV089A11 and CBV069I23) with a physical size of no more than 200 kb (average of 90 kb/cM). The fact that these markers were spaced at 2.2 cM and were located on two overlapping BACs could be indicative of a recombination hotspot and/or a fiber elongation gene-rich region. In comparison, two fiber EST markers were anchored in contig 2503 and co-segregate (cM = zero). In another case, there are two markers in contig 941 with a physical distance of no more than 250 kb and a genetic distance of 14.2 cM (ratio = 17 kb/cM). While more data is needed to estimate an accurate genome-wide ratio of genetic and physical distances, results from the comparison of these three contigs demonstrate the variation often observed in genetic to physical distance ratios and why it is so important to develop integrated genomic resources.

The choice of genotype is critical to the usefulness of any integrated genomic resource. In the case of cultivated cotton, the type and size of mapping populations are critical to obtain accurate genetic information. Based on available genetic maps, it is then necessary to select the appropriate genotype(s) from which to develop supporting genomic resources. As we noted above, among the 24 published genetic maps for cotton, genetic distances for most markers are variable due to differences in mapping populations. Cotton geneticists have used populations based on F_2_, F_2:3_, RIL, BC_1_, and DH in addition to different population sizes. Although a DH population has the advantage of being a permanent population providing many advantages in genetic map construction, it takes a lot of time and labor to develop a large DH population with semigamy structure. Currently, the RIL population using the Upland cotton genetic standard TM-1 (191 lines) is considered to be the best choice for genetic map construction. In fact, more research groups are using this population to facilitate data analysis and interpretation. It is especially beneficial that the current physical mapping effort in Upland cotton uses TM-1 as a DNA donor for the BAC libraries. The combination of genetic, physical, and cytogenetic information from TM-1 makes the cotton genome data more accurate and valuable.

### Chromosome coverage considerations

Even though all the publicly available DNA markers were collected and used for BAC library screening, there still remain gaps between the 220 and 115 contigs on chromosomes 12 and 26 with a coverage of 74 and 34 Mb, respectively, that remain unanchored. By using standard genome coverage calculations, at least a 10X haploid coverage is needed to represent about 95% of the genome [27] and a 20X coverage is needed to represent approximately 98% of the genome [28]. It is anticipated that an increase in clone coverage will aid in contig gap closure, particularly if alternative cloning enzymes are used.

In plants, many tandem repeats have been localized to specific chromosomal regions such as centromere, telomere, or heterochromatin by *in situ *hybridization, making them excellent landmarks for studying chromosome structure, function, and evolution. Telomere regions have been mapped using repetitive sequences in tomato [29], barley [30], and rice [31]. In cotton, a chromosome-specific tandem repeat 572-bp B77 was mapped to a single 550 kb Sal/I fragment in the Dt subgenome chromosome D04 of tetraploid cotton. FISH data showed that it was close to telomere region although not in the telomere region [32]. Thus, more clones are needed to fill the gaps and more repeat specific repetitive markers are needed to identify centromere and telomere regions of cotton chromosomes. It is likely that telomeric and centromeric BACs are represented in the available BAC libraries; it is just a matter of identifying them.

### Strategy to construct a genome-wide physical map of Upland cotton

In this report, we present a strategy using four steps to construct an integrated map of one pair of homeologous cotton chromosomes (12 and 26) in a complex allotetraploid plant genome. The first step was to collect all genetically mapped markers with associated DNA sequence and design overgo primers for BAC library screening. The second step was to screen the Upland cotton BAC libraries and to obtain positive BACs. The third step was to fingerprint the BACs and to assemble them into contigs. And the last step was to integrate unmapped EST unigenes onto the contigs providing a significantly enhanced level of map annotation. Detailed physical maps of the horse Y chromosome [33] and Papaya Y chromosomes [34] were constructed by the use of a similar strategy. The goal of this study was to test the feasibility of this approach in a complex polyploid genome where it is necessary to differentiate and separately characterize homoeolgous sets of chromosomes associated with different sub-genomes. Our results indicate that this is possible and we are now in the process of constructing an integrated physical map for the whole genome of Upland cotton. Our results also suggest that additional genetic markers and an increase in BAC library coverage would facilitate gap closure and the mapping of structurally important repetitive regions of chromosomes. However, positive results were obtained with existing resources as to contig allocation between homeologous chromosomes. Contigs that were not anchored or were mapped ambiguously to multiple chromosomes could eventually be assigned to individual chromosomes by additional BAC-derived SSR markers and SNP markers [35]. In rice, a fine-scale physical map of chromosome 5 was constructed using this approach [36]. Construction of an integrated physical map for an individual homoeologous chromosome pair in Upland cotton lays a foundation for many genomic applications, including eventual sequencing and annotation of the entire complete Upland cotton genome [37].

## Conclusion

This integrated genomic map describes the first pair of homeologous chromosomes of an allotetraploid plant in which BAC contigs were identified through the use of chromosome-specific probes and locus-specific genetic markers. The approach used in this study should prove useful in the construction of genome-wide physical maps for other polyploid plant genomes including Upland cotton; EST unigenes could be integrated into the BAC contig map to construct transcript map of cotton by overgo hybridization and sequence comparison, and thus gene-rich islands could be identified for function genomics.

## Methods

### BAC libraries

Two TM-1 BAC libraries were used in the study and were constructed at Texas A&M University with the USDA-ARS [10,11] using partial digestions with the restriction enzymes *Bam*HI and *Hind*III. The *Bam*HI library is cloned into a BAC-based binary plant transformation vector (BIBAC vector; pCLD04541) while the *Hin*dIII library was cloned using a standard BAC vector (pBeloBAC11). The *Bam*HI library contains 76,800 clones with an average insert size of 130 kb, and covering 4.4 haploid genome equivalents. The *Hind*III BAC library contains 76,800 clones with an average insert size of 152 kb. The third BAC library used in this study was constructed from the Upland cotton cultivar Maxxa using *Hin*dIII, at the Clemson University Genomics Institute [12] and contains 129,024 clones with an average insert size of 137 kb providing ~8X coverage. The Maxxa BAC library was partially end-sequenced (~50,000 reads) and mined for putative SSRs [7]. BAC clones associated with SSR markers located to chromosomes 12 and 26 were obtained from the library and included in fingerprinting. High-density colony filter arrays were prepared using a Biomek 2000 robotic workstation equipped with a high-density replicating system (HDR) (Beckman Coulter Inc., Fullerton, California). Each filter was gridded with 1,536 BAC clones using a 4 × 4 matrix pattern with a 384-pin HDR tool. Filters were incubated and processed as described by Woo [38].

### Overgo probe design and hybridization

All marker associated EST sequences were assembled into contigs using Sequencher 4.2 [39] (Gene Codes Corporation, Ann Arbor USA) to reduce redundancy. Sequence from each contig was masked to eliminate known repetitive regions using the RepeatMasker [40] and then entered into the Overgo 1.02i program to design overgo primers,[41,42]. Only one overgo probe was designed for each sequence contig. Each overgo sequence was examined to ensure that it contained sufficient sites for labeling by ^32^P-dATP and -dCTP (preferably at least 50% of the sequences are G and C). If fewer than 4 G-C bases occurred in the 8-bp overlap region, the length of overlap was increased to 10 bp to insure stable association between the two oligonucleotides. If it was still fewer than 4 G-C bp, no overgo probe was designed from this sequence. Pre-hybridization and hybridization followed the protocol as Cai [41]. Positive clones were recorded and re-arrayed into new 96-well plates for fingerprinting.

### BAC fingerprinting and contig assembly

The DNA of positive BAC clones was isolated with the PerfectPrep BAC 96 DNA purification kits (Brinkman Instruments, Inc). About 300–600 ng of the BAC DNA was used in the digestion and labeling reaction. The clones were digested with three enzymes (*Hind*III, *Bam*HI, and *Hae*III) and labeled with fluorescence dye NED or HEX (Applied Biosystems). Labeled fragments were separated in ABI 3100 DNA Analysis Machines and sizes of the DNA fragments were collected by GeneScan v3.70 in a range from 35 to 500 bases [27]. The BAC contigs were assembled and edited using Finger Printed Contigs, FPC version 8.5[28]. Contigs were assembled by: 1) clones from chromosome 12 specifically; 2) clones from chromosome 26 specifically; 3) clones from both chromosomes 12 and 26. Contigs from the three assemblies were compared and cross-verified.

### Contig analysis

Contigs with less overlap but with more than two neighboring markers in each contig were merged into one contig. Additional merges were made between contigs according to consistent genetic marker data if supported by fingerprint overlaps with probability scores of better than 1e-10 [43]. To sort contigs into subgenomes and to assign them to individual chromosomes, two strategies were employed. The first strategy was to use the subgenome-specific markers to separate contigs to subgenome At or Dt. The second strategy was to use linkage group and locus-specific markers to assign contigs to individual chromosomes. Several genetic markers specific to the subgenome At and Dt of tetraploid cotton were previously developed via representational difference analysis RDA [44]. In a later study, both the markers and their development method proved useful in developing At and Dt subgenome-specific markers in Upland cotton [45].

Contigs obtained by hybridization in this report were compared and verified with those from the preliminary genome-wide physical contig map (unpublished). To increase coverage of the two chromosomes, equal amounts of DNA for each positive BAC clone identified by non-repetitive markers were pooled for each chromosome and the pools used as bulk probes to screen the three libraries. Overlapping, newly identified clones from the genome-wide physical map were added for chromosomes 12 and 26.

**Chromosome Homoeology rate calculation **for genetic markers based, homoeologous rate was calculated by compare the sequences using the formula: homologous rate = 2 × shared sequences/(marker sequences in chromosome 12 and 26) × 100%; for contig based, homologous rate = 2 × length of shared contigs/(total length of contigs in chromosomes 12 and 26) × 100 %

### Anchoring EST unigenes

Overgo hybridization was also used to anchor cotton EST unigenes to the chromosomes that were not associated with genetic markers. A total of 51,107 cotton unigenes were downloaded from Cotton EST unigene database [46]. The Blast program "blastall" was downloaded from NCBI [47] and used to annotate the sequence. The criterion for sequence match, expected value E = 1e-30, was used to perform the blast analysis.

## Authors' contributions

ZX participated in the experiment design, genetic marker collection, overgo design, BAC screening, contig assembly and verification, EST unigene anchoring, identification of gene-rich islands, perl script writing for data analysis, and manuscript drafting. RJK and JZY initiated and supervised all aspects of the project including the experiment design and implementation as well as data analysis and manuscript revisions. GS participated in BAC screening, EST unigene anchoring, perl script writing for data analysis. JC participated in BAC screening. JY loaded and maintains the project data in the database. SY participated in the coordination and implementation of the project. JT provided Maxxa BAC clones as well as contributed to analyses of the data and revisions of the manuscript. All authors read and approved the final manuscript.

## Supplementary Material

Additional file 1 Marker information of chromosomes 12 and 26. The data provided all the markers' information of the chromosomes 12 and 26. Click here for file

Additional file 2 Contigs for chromosome 12 and clones shared between chromosomes 12 and 26. The dataset listed all the contigs for chromosome 12 and clones shared between chromosomes 12 and 26 Click here for file

Additional file 3 Contigs for chromosome 26. This dataset listed all the contigs for chromosome 26 Click here for file

Additional file 4 Homology rates of the 13 pairs of the chromosomes. This data provided the homology rates of the 13 pairs of the homoeologous chromosomes. Click here for file

Additional file 5 Unigenes anchored to chromosome 12. This dataset listed all the EST unigenes anchored on chromosome 12 Click here for file

Additional file 6 Unigenes anchored to chromosome 26. This dataset listed all the EST unigenes anchored on chromosome 26 Click here for file

Additional file 7 Integrated genetic, physical and transcript map of chromosome 12. This figure showed the whole picture integrated genetic, physical and transcript map of chromosome 12. Three columns are displayed in the figure (left, middle and right). Left column shows the fiber EST unigenes anchored to the chromosome 12; Middle column shows the genetic map, and right column shows the contigs assembled from the positive clones to the genetic markers. The markers in black were used as backbone markers that were derived from an F2 mapping population (G. hirsutum race "palmeri" and G. barbadense acc. "K101); markers in red (MUSB) were from BAC-end sequence and genetic distance was from the RIL mapping population (G. hirsutum TM-1 × G. barbadense 3–79); markers in green (TMB) were from BAC subcloned sequence and mapped by the TM-1 × 3–79 RIL population; the blue markers were from BC1 mapping population ('Guazuncho 2' × 'VH8-4602'). Markers in pink at the bottom of the figure were from BC1 mapping population (TM-1 × (TM-1 × Hai7124). CUN stand for Cotton Unigene Number that was used in the original paper [16]. Click here for file

Additional file 8 Integrated genetic, physical and transcript map of chromosome 26. This figure showed the whole picture integrated genetic, physical and transcript map of chromosome 26. The legends are same as described for Additional file 7. Click here for file
