# Supplementary figures and images for: An integrated genetic and physical map of homoeologous chromosomes 12 and 26 in Upland cotton (G. hirsutum L.)
Source: BMC Genomics. 2008 Feb 28;9:108. doi: 10.1186/1471-2164-9-108 (PMC2270834; doi:10.1186/1471-2164-9-108)

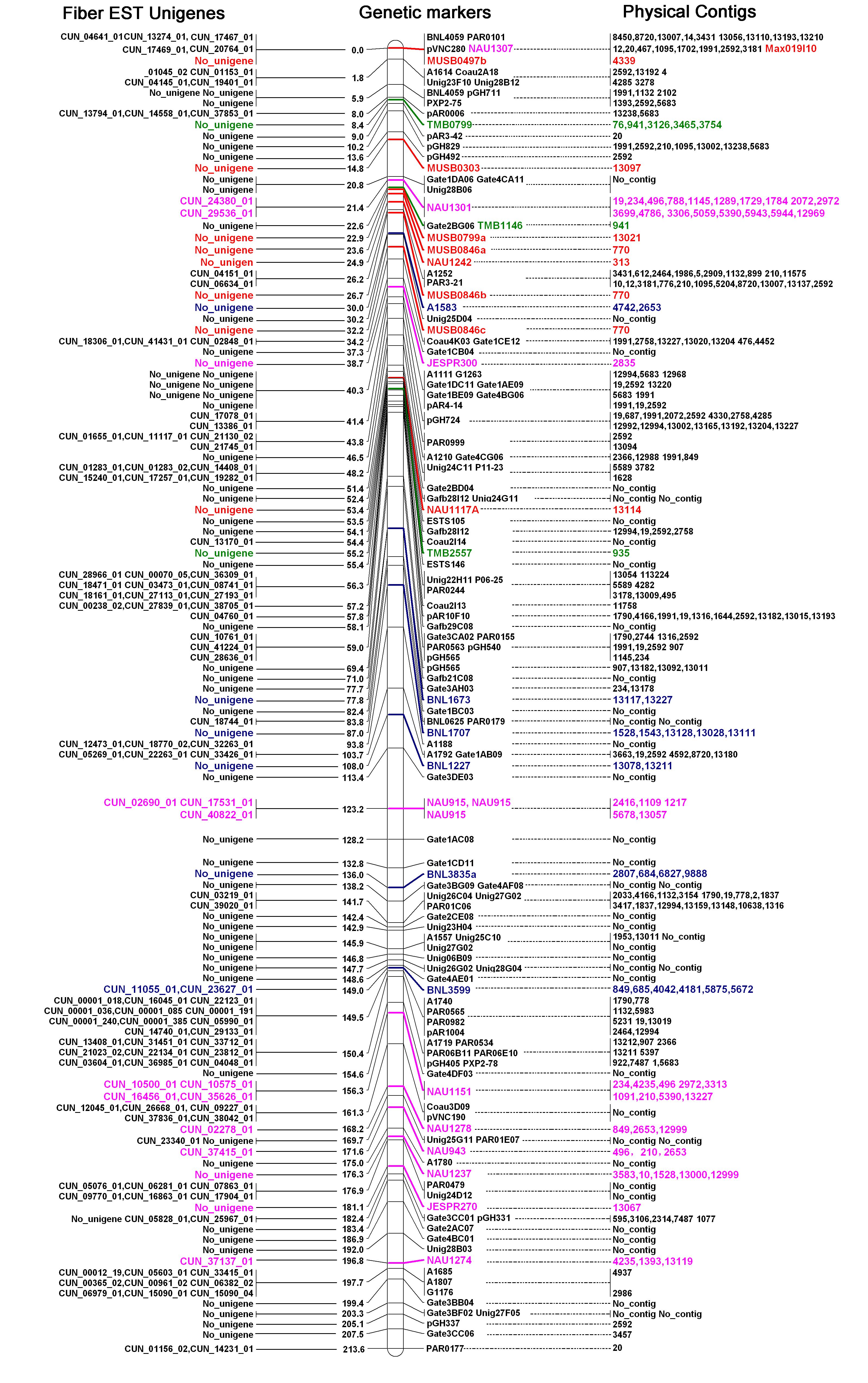

Supplement: Additional file 7 — Integrated genetic, physical and transcript map of chromosome 12. This figure showed the whole picture integrated genetic, physical and transcript map of chromosome 12. Three columns are displayed in the figure (left, middle and right). Left column shows the fiber EST unigenes anchored to the chromosome 12; Middle column shows the genetic map, and right column shows the contigs assembled from the positive clones to the genetic markers. The markers in black were used as backbone markers that were derived from an F2 mapping population (G. hirsutum race "palmeri" and G. barbadense acc. "K101); markers in red (MUSB) were from BAC-end sequence and genetic distance was from the RIL mapping population (G. hirsutum TM-1 × G. barbadense 3–79); markers in green (TMB) were from BAC subcloned sequence and mapped by the TM-1 × 3–79 RIL population; the blue markers were from BC1 mapping population ('Guazuncho 2' × 'VH8-4602'). Markers in pink at the bottom of the figure were from BC1 mapping population (TM-1 × (TM-1 × Hai7124). CUN stand for Cotton Unigene Number that was used in the original paper [16]. [file 1471-2164-9-108-S7.jpeg]

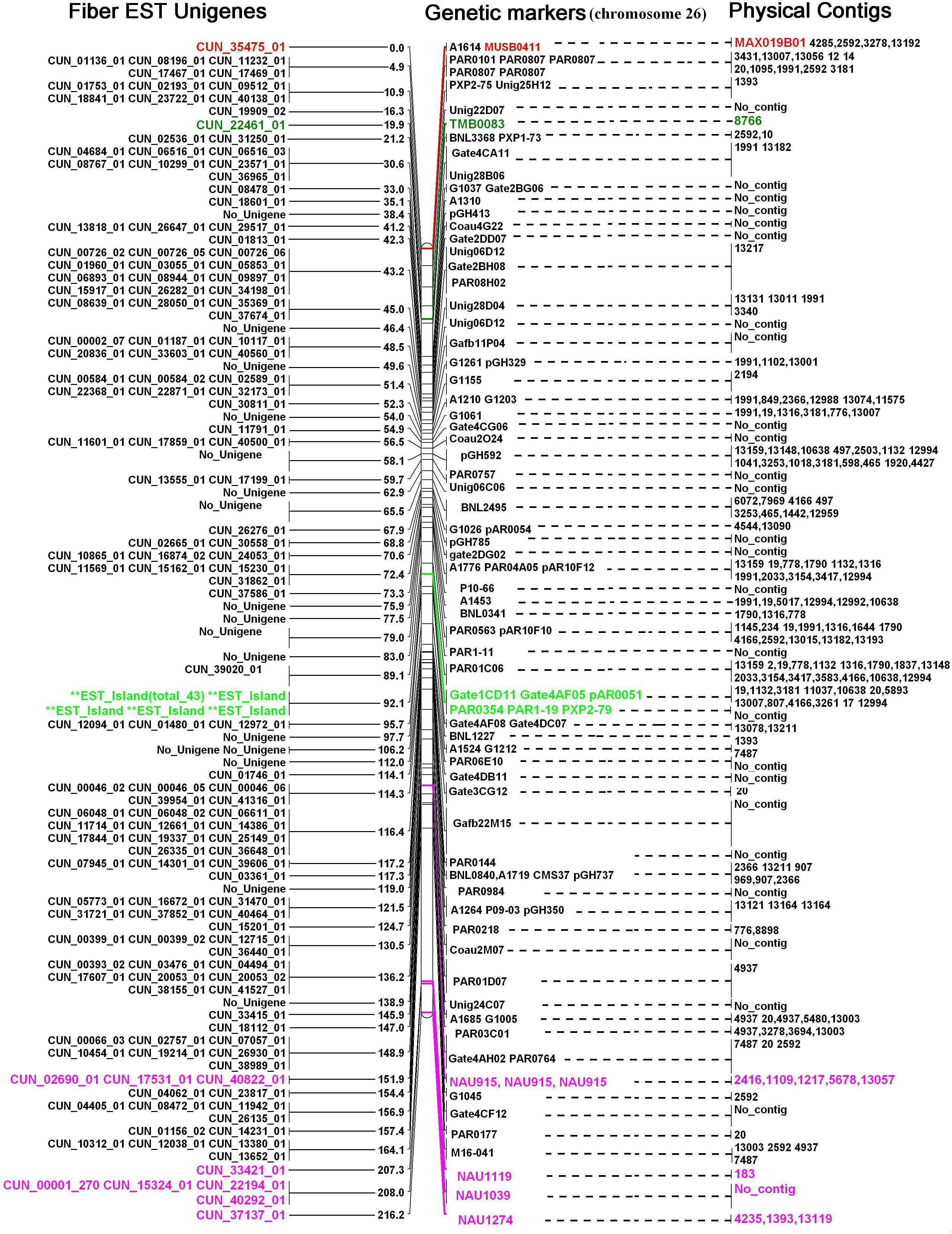

Supplement: Additional file 8 — Integrated genetic, physical and transcript map of chromosome 26. This figure showed the whole picture integrated genetic, physical and transcript map of chromosome 26. The legends are same as described for Additional file 7. [file 1471-2164-9-108-S8.jpeg]
